# Withdrawal Notice: SAMD1 attenuates antiphospholipid syndrome‐induced vascular injury and pregnancy complications

**DOI:** 10.1002/iid3.761

**Published:** 2022-12-31

The following article, SAMD1 attenuates antiphospholipid syndrome‐induced vascular injury and pregnancy complications; doi/10.1002/iid3.678, published online on August 17, 2022, in Wiley Online Library (wileyonlinelibrary.com), has been withdrawn by agreement between the journal Editor in Chief, Dr Marc Veldhoen, and John Wiley & Sons, Ltd. The withdrawal has been agreed due to an editorial office error that led to the publication of the article without peer review.

